# Three-Dimensional Kinetic Adaptations of Gait throughout Pregnancy and Postpartum

**DOI:** 10.1155/2015/580374

**Published:** 2015-09-30

**Authors:** Marco Branco, Rita Santos-Rocha, Filomena Vieira, Liliana Aguiar, António Prieto Veloso

**Affiliations:** ^1^CIPER Interdisciplinary Centre for the Study of Human Performance, Faculty of Human Kinetics, University of Lisbon, Estrada da Costa, Cruz Quebrada-Dafundo, 1499-002 Lisboa, Portugal; ^2^ESDRM-IPS Sport Sciences School of Rio Maior, Polytechnic Institute of Santarém, Avenida Doutor Mário Soares, Rio Maior, 2040-413 Santarém, Portugal; ^3^FMH-UL Faculty of Human Kinetics, University of Lisbon, Estrada da Costa, Cruz Quebrada-Dafundo, 1499-002 Lisboa, Portugal

## Abstract

Biomechanical adaptations that occur during pregnancy can lead to changes on gait pattern. Nevertheless, these adaptations of gait are still not fully understood. The purpose was to determine the effect of pregnancy on the biomechanical pattern of walking, regarding the kinetic parameters. A three-dimensional analysis was performed in eleven participants. The kinetic parameters in the joints of the lower limb during gait were compared at the end of the first, second, and third trimesters of pregnancy and in the postpartum period, in healthy pregnant women. The main results showed a reduction in the normalized vertical reaction forces, throughout pregnancy, particularly the third peak. Pregnant women showed, during most of the stance phase, medial reaction forces as a motor response to promote the body stability. Bilateral changes were observed in hip joint, with a decrease in the participation of the hip extensors and in the eccentric contraction of hip flexors. In ankle joint a decrease in the participation of ankle plantar flexors was found. In conclusion, the overall results point to biomechanical adjustments that showed a decrease of the mechanical load of women throughout pregnancy, with exception for few unilateral changes of hip joint moments.

## 1. Introduction

During the normal development of pregnancy, women experience a variety of morphological and physiological changes, as well as a continuous increase in body weight [1, 2]. However, the dynamics of the load that women experience and how this weight gain influence everyday tasks, such as walking, are not fully described. Walking is widely used not only as a mean of locomotion, but also as a mode of exercise, especially recommended throughout pregnancy and without contraindications for pregnant women [3].

The ground reaction forces (GRF) have been widely studied in the characterization of walking; however, only in the last two decades these parameters were studied in pregnant women. The importance of these parameters to investigate the stability and possible causes of falls in pregnant women has been the focus of recent studies. Lymbery and Gilleard [4] concluded that in late pregnancy the mediolateral GRF tended to be increased in a medial direction along with a wider step width, suggesting that women may adapt their gait to maximize stability in the stance phase of walking and to control mediolateral motion. Branco et al. [5], in a previous study, analyzed the tridimensional adaptations of kinetic parameters between the second and third trimesters of twenty-four pregnant women and with a control group. Most of the results reveal significant changes between pregnancy stages and nonpregnant group, reporting a decrease of the mechanical load of pregnant women, specifically with a lower magnitude in the third peak of vertical GRF and in the second peak of anterior component of GRF, and most of the stance phase has a medial GRF. According to Abu Osman and Ghazali [6] and McCrory et al. [7] no changes were found for GRF in the collected groups. However, in most studies only the last stages of pregnancy were considered (with the exception of Abu Osman and Ghazali [6] and Inanir et al. [8]), not getting clearly defined if there is any influence of pregnancy condition on these variables, and if these changes happen gradually from the beginning of pregnancy. Such as the GRF, the joint kinetics is mostly studied in the last two trimesters of pregnancy or between the end of pregnancy and postpartum (with exception for the study of Huang et al. [9]). Foti et al. [10] regarding this analysis, which reported significant increases in hip and ankle kinetics during pregnancy). Their findings show that during pregnancy there may be an increased demand placed on the hip abductors, hip extensors, and ankle plantarflexors muscles during walking. Branco et al. [5] analyze the tridimensional adaptations of kinetic parameters between the second and third trimesters of twenty-four pregnant women and with a control group. Most of the changes reveal significant changes between pregnancy stages and nonpregnant group. In the joint moments they found a decrease in hip flexors, knee extensors, ankle dorsiflexors, and ankle evertors' moment and an increase in the participation of hip external rotators during loading response phase of pregnant women. In the joint powers they found significant decreases in the absorption of mechanical energy of hip flexors and significant decreases in the production of mechanical energy of the hip abductors and in the ankle plantarflexors. An increase in the production of mechanical energy was found for hip extensors and hip external rotators. Huang et al. [9] compared the gait pattern of nonpregnant and pregnant women divided into three groups, respectively: 12 weeks, 13–28 weeks, and 29–40 weeks of gestation, which were tested only once. They reported significant differences between the pregnant and nonpregnant women, especially in knee abduction angle, knee and hip internal rotation angles, hip extension moment, and hip power. Also, as gestational age increased, the pregnant group increased hip extension moment, decreased knee extension moment, increased knee adduction moment, and decreased ankle plantar flexion moment. It is not clear however, in the different studies (with the exception of Branco et al. [5]), if these changes occur bilaterally, occur only unilaterally, or correspond to average values between sides.

Thus, the main purpose of this study was to assess the gait kinetics, in order to check if there are any changes in the dynamics of the load of women from the beginning of pregnancy until the postpartum period. To achieve the main objective, it was necessary to describe and quantify the kinetic variables, regarding GRF, joints moment of force, and joints power, of ankle, knee, and hip, during gait, at the end of the first, second, and third trimesters of pregnancy and in the postpartum period.

We tested the hypothesis that the GRF, joints moment, and joints power parameters exhibit deviations along the pregnancy and are associated with this special phase of life, which are recovered in the postpartum period.

## 2. Materials and Methods

### 2.1. Subjects

Eleven pregnant women, aged between 32 and 37 years (33.20 ± 1.62 years), with height between 1.60 and 1.73 meters (1.64 ± 0.04 m), with no history of foot, ankle, knee, musculoskeletal, and neuromuscular trauma or disease, participated in this study. The participants have the following characteristics of weight and weeks of gestation (wk) in the four periods of data collection: (1) first trimester: 61.1 ± 6.6 kg and 14.2 ± 2.4 wk; (2) second trimester: 66.6 ± 8.5 kg and 27.3 ± 1.0 wk; (3) third trimester: 71.0 ± 8.0 kg and 36.3 ± 0.9 wk; and (4) postpartum period: 62.4 ± 7.4 kg and 20.6 ± 5.2 weeks after birth.

The participants were recruited via direct contact and flyers placed in gym and health centres and have volunteered to participate in the study. None of the participants had contraindication to physical exercise. All subjects gave written informed consent prior to the participation in the study.

### 2.2. Data Collection and Processing

The study was approved by the ethical committee of the Faculty of Human Kinetics (University of Lisbon) and data were collected at the Laboratory of Biomechanics and Functional Morphology, in four periods: during the later stages of the first, second, and third trimesters of gestation and in the postpartum period. Before performing the motor task, anthropometric data was measured (weight and height), according to the International Society for the Advancement of Kinanthropometry (ISAK) standardized measurement protocol [11] by ISAK certified anthropometrists, to calculate the body segment masses and inertia moments.

In order to collect static and dynamic data trials, spherical reflective markers were placed on the skin on both sides of the lower body with double-sided adhesive tape. Markers setup is in agreement with the suggestion of Cappozzo et al. [12], for lower limb segments, and CODA (Charnwood Dynamics Ltd., Leicestershire, UK) protocols for model of the pelvis segment.

The motor task was to walk barefoot a distance of 10 meters between two points, in a straight line in both directions at a natural and comfortable speed, during 3 minutes, stopping and starting the path in the opposite direction. The floor had no specific patterns or irregularities, and the participants had no knowledge about the location of force platforms, which were placed in the middle of the defined distance. Participants were allowed to get familiar with the laboratory system and no fatigue occurrence was reported.

Planar motion of the hip, knee, and ankle joints was calculated with Visual 3D (V3D) software (C-Motion Inc., Germantown, USA) by a computational procedure implementing the dot product between the skeletal segments articulated by these joints.

Kinematic data were collected through twelve infrared high-speed cameras (Oqus-300, Qualisys, Sweden) at a rate of 200 Hz, and kinetic data were collected with two Kistler force platforms (Kistler AG, Winterthur, Switzerland) of 0.60 m × 0.40 m and one AMTI platform (Advanced Mechanical Technology, Inc., Watertown) of 0.90 m × 0.60 m, at a rate of 1000 Hz. The capture hardware was connected to Qualisys USB Analog Acquisition interface in order to synchronize kinetic and kinematic data with software Qualisys Track Manager (QTM; Qualisys AB, Gothenburg, Sweden). Both data sequences were recorded in the same file. The system was calibrated by wand type, with an exact wand length of 751.4 mm moved randomly across the recorded field, before the data collection of each participant. Calibration was accepted if the standard deviation of the wand's length measures was below 0.5 mm. Cameras were positioned statically to minimize light reflection artefacts and to allow recording of at least two consecutive walking cycles, defined as the time between two consecutive initial ground contacts of the heel strike with each foot. The last four cycles within the period of three minutes performed by each participant were considered for the analysis.

A three-dimensional (3D) analysis was performed including both sides of the body. The trajectory of the reflective markers was smoothed using a Butterworth low-pass filter with a 10 Hz cutoff. Collected data were interpolated using a Cubic Spline Interpolation as suggested by Robertson et al. [13], for a maximum of 10 frames gap, and filtered with a Butterworth digital low-pass filter, at 10 Hz cutoff frequency for kinematic and kinetic data, as suggested by Robertson and Dowling [14]. All data were normalized in time. The setup of markers used is described in our previous study [15].

Considering the four time phases in analysis, kinetic pattern curves of the three components of the GRF were normalized to units of bodyweight and were estimated relative to the total time of contact of the foot on the ground. These data curves and peak values were estimated for left and right sides, with Visual 3D software (C-Motion, Inc., Germantown, USA).

The net joint moments and powers of the ankle, knee, and hip were calculated using an inverse dynamics approach, considering an *XYZ* Cardan sequence for joint angles, and resolved for the proximal segment's coordinate system [16] in Visual 3D software. For kinetic parameters, initial foot contact was collected at the time corresponding to first contact of the foot on the floor, while final contact was collected with the last contact of the foot on the floor.

In the four periods under analysis, the following variables of the GRF curves were analyzed: three peaks characterize the vertical component, two peaks characterize the anterior-posterior component, and two peaks characterize the medial-lateral component.

The joints' moments' patterns were the following: the hip moment is characterized by two peaks in the sagittal plane, two peaks in the frontal plane, and one peak in the transverse plane. The knee moment is characterized by three peaks in the sagittal plane. The ankle moment is characterized by two peaks in the sagittal plane, one peak in the frontal plane, and two peaks in the transverse plane.

The joints' power patterns were the following: the hip power is characterized by two peaks in the sagittal plane, three peaks in the frontal plane, and two peaks in the transverse plane. The knee power is characterized by three peaks in the sagittal plane. The ankle power is characterized by two peaks in the sagittal plane, two peaks in the frontal plane, and two peaks in the transverse plane.

### 2.3. Statistical Analysis

All statistical procedures were conducted using IBM SPSS Statistics 22 software for Windows. Shapiro-Wilk normality tests were conducted and not assumed for all cases. The Mauchly's test of sphericity was performed before Repeated Measures (RM) analysis and sphericity was not assumed in all tests. For post hoc tests, the Bonferroni test was used based on Student's *t* statistic, adjusting the observed significance level for the fact that multiple comparisons were made. For variables and groups which did not commit all assumptions of RM analysis, the Friedman test was performed, as well as the Wilcoxon test in the case of pairwise analysis. In this case, Bonferroni confidence interval adjustment was applied to allow an adjustment to the confidence intervals and significance values for multiple comparisons.

## 3. Results

### 3.1. Ground Reaction Forces

**Figure 1:**
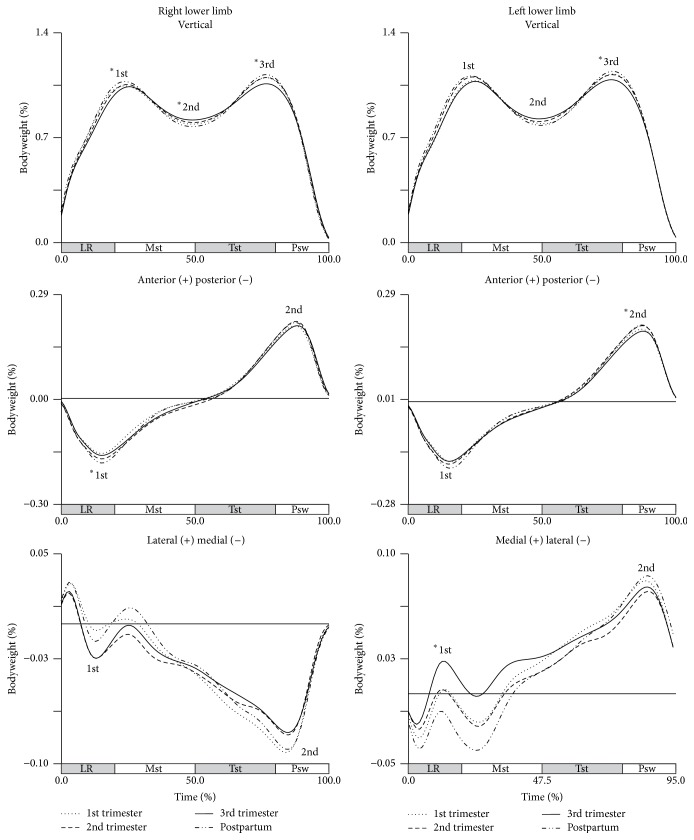
Mean values of ground reaction forces of each group (in bodyweight). Each line represents the later stages of first (dotted line), second (dashed line), and third trimesters (solid line) and of postpartum period (dash-dot-dot line). Mean vertical, anterior-posterior, and medial-lateral components of GRF, for right and left lower limbs. The curve peaks are indicated by numbers: 1st, 2nd, and 3rd, and (*∗*) points the significant differences.

Most of the components of the GRF are influenced by the stage of pregnancy, particularly in the three peaks of the vertical component, in the two peaks of the anterior-posterior component, and at the first peak of the medial-lateral component of GRF (Figure 1).

The descriptive statistics of the GRF can be found in Table 1. Our results suggest a decrease of GRF in late pregnancy.

The first and second peaks of vertical GRF show significant changes from third trimester to the postpartum period, emphasizing an increase of 4% in the braking peak and a decrease of 5% of bodyweight (BW) in the inverted peak for right lower limb. In both stances, a significant decrease of 5% BW in the third peak from the first to the third trimesters and an increase of about 7% BW from the end of pregnancy to postpartum period were observed, which correspond to a recovery to values above those recorded in early pregnancy. Also in propulsive peak, to the right stance, there is a decrease of 4% BW from second to third trimesters. In the anterior-posterior component of GRF, an increase of 2% BW from first trimester to postpartum period in the posterior direction (first peak) of the right lower limb and a decrease of 2% BW in the anterior direction (second peak) of the left lower limb were found which represent that at the end of pregnancy the participants apply less of their bodyweight of force against the floor in the loading response phase and in the preswing phase. The medial-lateral component of GRF decreases in the first peak, moving from a medial reaction to a lateral reaction in the third trimester to the postpartum period.

### 3.2. Joint Moments of Force

#### 3.2.1. Sagittal Plane

**Figure 2:**
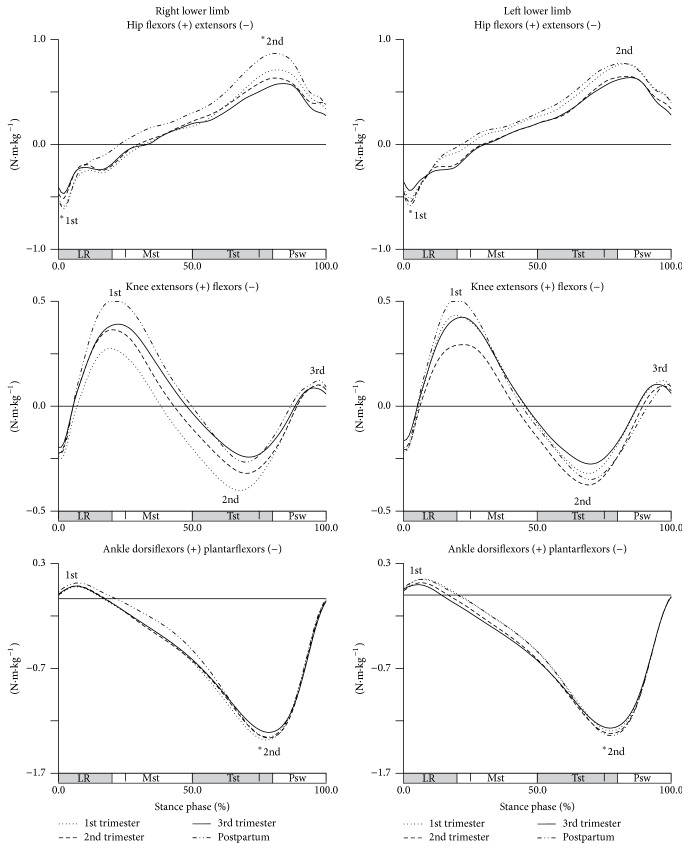
Joints moments in the sagittal plane during gait (in N·m·kg^−1^). Each line represents the later stages of first (dotted line), second (dashed line), and third trimesters (solid line) and of postpartum period (dash-dot-dot line). Mean joint moments of the hip, knee, and ankle, for right and left lower limbs. The curve peaks are indicated by numbers: 1st, 2nd, and 3rd, and (*∗*) points the significant differences.

A longitudinal effect of pregnancy in joint moments of force, in sagittal plane, was observed for some peaks of hip and ankle joints (Figure 2).

Descriptive statistics of the joints moment, and the significance levels of the pairs of collections with significant changes for sagittal, frontal, and transverse planes, are presented in Table 2.

The moment of force in hip joint shows a significant decrease of 0.128 N·m·kg^−1^ in the action of hip extensors, during loading response phase from first to third trimesters in the right lower limb and an increase of, respectively, 0.87 N·m·kg^−1^ and 0.154 N·m·kg^−1^ from first (left side) and third trimesters (both sides) to postpartum period, indicating that there is an opposite trend to what happens during pregnancy even from the beginning. In terminal stance phase, only for right side, there is a significant decrease of 0.156 N·m·kg^−1^ in the action of the hip flexors from the beginning to the end of pregnancy, and an increase of, respectively, 0.208 N·m·kg^−1^ and 0.285 N·m·kg^−1^ from the second and third trimesters to postpartum, similar to what happens at the beginning of the stance phase. No changes were found in knee moments of force during pregnancy and postpartum period. The ankle plantarflexors participation shows a significant decreasing of 0.082 N·m·kg^−1^ from first to third trimesters, in right ankle, and an increase of 0.075 N·m·kg^−1^ on their participation from late pregnancy to postpartum period, for left ankle.

#### 3.2.2. Frontal Plane

**Figure 3:**
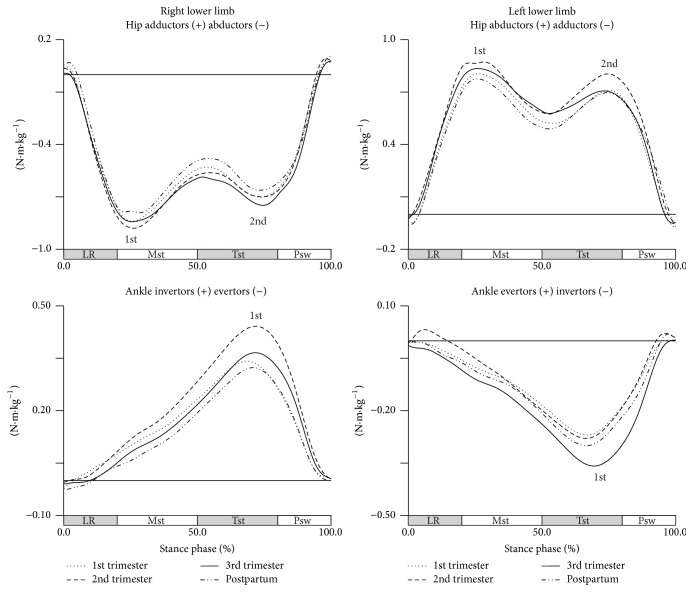
Joints moments in the frontal plane during gait (in N·m·kg^−1^). Each line represents the later stages of first (dotted line), second (dashed line), and third trimesters (solid line) and of postpartum period (dash-dot-dot line). Mean joint moments of the hip and ankle, for right and left lower limbs. The curve peaks are indicated by numbers: 1st and 2nd, and (*∗*) points the significant differences.

The longitudinal effect of pregnancy was not observed in frontal plane, and therefore no significant changes were found in any of the analyzed variables (Figure 3).

#### 3.2.3. Transverse Plane

**Figure 4:**
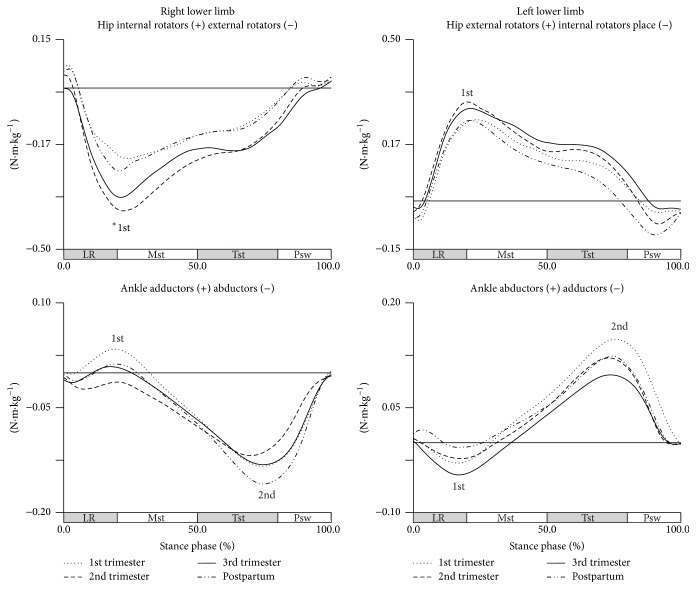
Joints moments in the transverse plane during gait (in N·m·kg^−1^). Each line represents the later stages of first (dotted line), second (dashed line), and third trimesters (solid line) and of postpartum period (dash-dot-dot line). Mean joint moments of the hip and ankle, for right and left lower limbs. The curve peaks are indicated by numbers: 1st and 2nd, and (*∗*) points the significant differences.

In transverse plane, the effect of pregnancy is only observable in hip joint for midstance phase (Wilks' *λ* = 0.201, *F*(3,8) = 10.573, *p* = 0.004) (Figure 4).

During this phase, there is an increase in the participation of the external rotators of the right hip throughout pregnancy, most pronounced between the first and second trimesters and less pronounced between the first and third trimesters, with increases in 0.204 N·m·kg^−1^ and 0.106 N·m·kg^−1^, respectively. From the second trimester to the postpartum period, the share of external rotators of the hip decreased significantly to values close to those found in early pregnancy.

### 3.3. Joint Power

#### 3.3.1. Sagittal Plane

**Figure 5:**
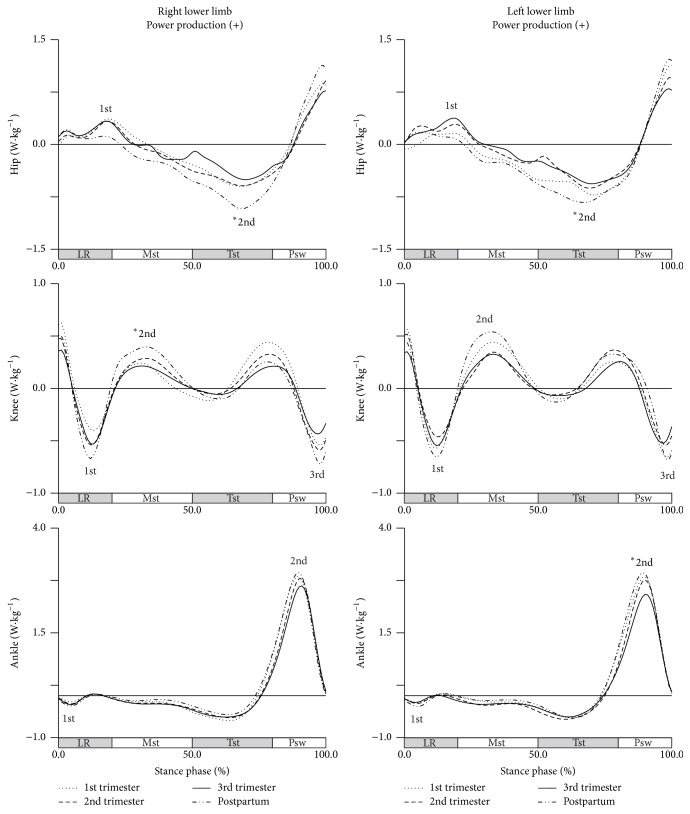
Joints power in the sagittal plane during gait (in W·kg^−1^). Each line represents the later stages of first (dotted line), second (dashed line), and third trimesters (solid line) and of postpartum period (dash-dot-dot line). Mean joint powers of the hip, knee, and ankle, for right and left lower limbs. The curve peaks are indicated by numbers: 1st, 2nd, and 3rd, and (*∗*) points the significant differences.

A longitudinal effect of pregnancy in joints power was observed for some peaks of hip and knee joints (Figure 5).

Table 3 shows the descriptive statistics of the joints power, and the significance levels of the pairs of collections with significant changes for sagittal, frontal, and transverse planes. In general, joints power in the postpartum period has similar values of early pregnancy, when compared to the values observed during late pregnancy.

The second peak of hip joint power shows a significant increase of 0.372 W·kg^−1^ and 0.257 W·kg^−1^, respectively, for right and left lower limbs, from third trimester to postpartum period, which indicates a lower execution of eccentric contraction of hip flexors during terminal stance phase in late pregnancy. The influence of pregnancy on knee joint was verified in the second and third peaks; however, a significant increase of 0.145 W·kg^−1^ was found only in the second peak between first trimester and postpartum period, which means that during midstance phase, pregnant women in early pregnancy did a minor concentric contraction of the knee extensors. In the power of ankle joint there were no significant changes found between collection phases.

#### 3.3.2. Frontal Plane

**Figure 6:**
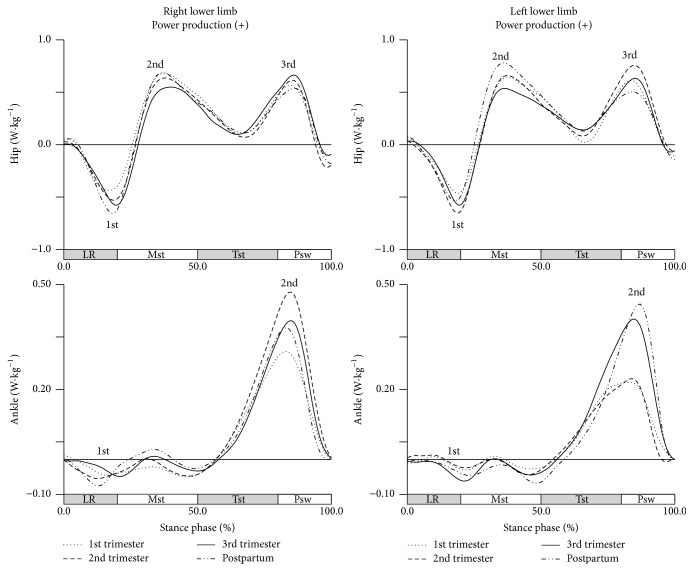
Joints power in the frontal plane during gait (in W·kg^−1^). Each line represents the later stages of first (dotted line), second (dashed line), and third trimesters (solid line) and of postpartum period (dash-dot-dot line). Mean joint powers of the hip and ankle, for right and left lower limbs. The curve peaks are Indicated by numbers: 1st, 2nd, 3rd, and 4th, and (*∗*) points the significant differences.

The longitudinal effect of pregnancy was not observed in the frontal plane, except for the first peak power of the left hip joint (Figure 6).

However, the post hoc tests show that this influence does not cause significant changes between pairs of collections.

#### 3.3.3. Transverse Plane

**Figure 7:**
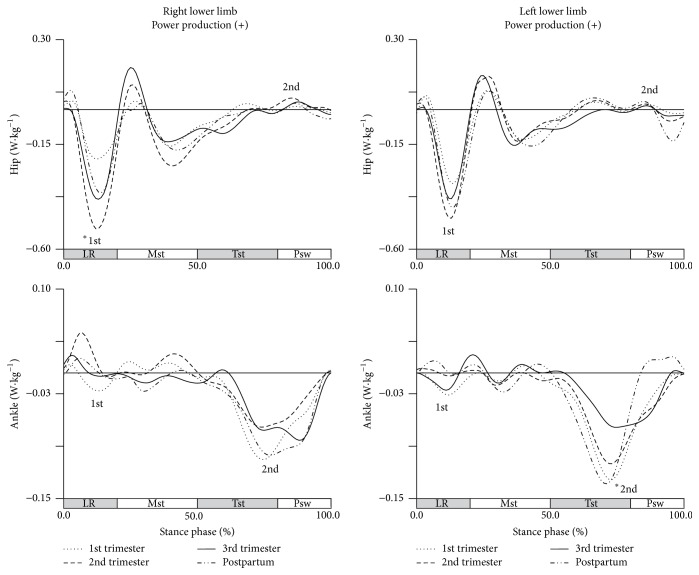
Joints power in the transverse plane during gait (in W·kg^−1^). Each line represents the later stages of first (dotted line), second (dashed line), and third trimesters (solid line) and of postpartum period (dash-dot-dot line). Mean joint powers of the hip and ankle, for right and left lower limbs. The curve peaks are indicated by numbers: 1st, 2nd, 3rd, and 4th, and (*∗*) points the significant differences.

A longitudinal effect of pregnancy in joints power of transverse plane was observed for some peaks of hip and ankle joints (Figure 7).

In right hip joint, an increase of 0.365 W·kg^−1^ in eccentric contraction of the external rotators of the hip was observed between first and second trimesters of pregnancy, during loading response phase. The left ankle joint shows a significant decrease of around 0.06 W·kg^−1^ in the ankle abductors, from the first and second trimesters to late pregnancy, which represents a lower eccentric contraction of these muscles during terminal stance phase.

## 4. Discussion

This study aimed to analyze the biomechanics of gait during pregnancy and postpartum period in a longitudinal perspective, in order to understand if there are any changes in the kinetic parameters of gait between collection phases. While other studies did not found any changes in the vertical or anterior components of GRF [4, 6, 7], in this study the quantification of vertical GRF has shown a decrease in the braking propulsive peaks from late pregnancy to postpartum period where it has values similar to those found in the first two trimesters of pregnancy. This result suggests that downward acceleration for the advancement of the body of the pregnant women and respective force production in the transmission of weight are smaller in late pregnancy [17]. In the medial-lateral component, it was found that in late pregnancy women have more medial reaction for left lower limb, similarly to the results also found by Takeda et al. [18] and our previous study [5] particularly during the loading response phase, which means that participants maintain the medial force during most of the stance phase. Few studies have found significant changes in the medial reaction forces, but many authors make reference to a greater instability in the frontal plane of the pregnant woman (e.g., [4, 5, 7]), for which these results indicate the motor response to this instability, thereby promoting greater body stability. The fact that the changes only happen in one of the lower limbs shows that the compensations derived from imbalances can happen only in one of the limbs to maintain balance and reinforces the need to make analyzes of the two sides.

In the sagittal plane, the kinetics of the hip joint has a significant reduction in the participation of the hip extensors during the loading response phase, which is highly associated with pregnancy. The participation of the hip extensors is related with the acceptance and support of the weight, which may be adversely affected by the reduced contribution of these muscles as the pregnancy progresses. At the terminal stance phase a reduction in the participation of the right hip flexors was established, also accompanied by a decrease in eccentric contraction of these muscles. In this phase, the extension of the thigh is promoted by inertia and gravity [19], and these results suggest less control in this motor action by pregnant women. In fact, the main objective of the hip joint muscles during the stance phase is the stabilization of the trunk [17], function that is affected, taking into account the majority of the results found.

In our previous study [5] there was a significant decrease in knee joint moments between the group of pregnant women and the group of nonpregnant women, showing that changes in the knee joint moments happen until the end of the first trimester. This is not observed in the present study, where the participation of the knee joint flexor and extensor remain similar throughout pregnancy. However, the variation of mechanical energy in these muscle groups has been influenced by pregnancy, with a significant reduction in the production of mechanical energy of the right knee extensors during midstance phase. The main function of the kinetics of the knee during this phase is to stabilize lower limbs when the total body weight is transferred for a single limb support, which in this case might be compromised. The kinetics of the ankle is influenced by pregnancy in participation of the plantar flexors during the preswing phase, with a decrease throughout pregnancy and a recovery of that participation in postpartum period. Two indications can be drawn from these results: the first is that the acceleration of the limb forward will be lower in late pregnancy and the second is that this participation is fully recovered after delivery.

Other studies reported some changes in the joint moments for the frontal plane, particularly an increase of the hip joint moments [10] (tested only between third trimester and postpartum) and the knee joint moments [9] and a decrease of ankle joint moments [5]. In this study no changes were found throughout pregnancy or postpartum. However, we found the influence of pregnancy on the absorption of mechanical energy in the hip abductors during late loading response phase, without a specific change between collected phases. One possible reason for the absence of any changes in the frontal plane may be revealed by the descriptive statistics of these variables, which relates to the fact that they present the highest variability (standard deviation) between the analyses of the planes of motion.

Only our previous study [5] reports changes during pregnancy in the transverse plane kinetics. In this study, during the loading response phase, significant increases were found in the participation of the right hip external rotators and eccentric contraction of these muscles, particularly in the second half of the pregnancy. This action is responsible for deceleration of the pelvis rotation, which aids in the advancement of the contralateral thigh [17], indicating that during pregnancy there is an increased control in advance of the contralateral limb. During the terminal stance phase, a decrease in eccentric contraction of the left ankle abductors along the pregnancy was observed, which indicates less control in the position of the left foot during the first moments of heel rise. The hypothesis of this study, where pregnancy is associated with kinetic adaptations, is partially confirmed by many of the kinetic parameters, for the three planes of motion, only partially because changes were found in many kinetic variables, but only in few of them the changes happen bilaterally and the recovery of these parameters was not confirmed in all cases, especially in the transverse plane. This result indicates that some situations of body instability might be maintained in the postpartum, suggesting the importance of physical activity, in order to promote the full recovery of the body stability. In the sagittal plane, for the vast majority of the parameters with significant changes, a recovery to values similar to those found in early pregnancy was observed, confirming the hypothesis raised.

According to Forczek and Staszkiewicz [20], the pregnant woman has two main strategies for adjustment of biomechanical parameters: first to increase the body stability, and second to reduce the energy expenditure. Based on our results, the second approach seems to be adopted in respect to the kinetic parameters. However, most studies on kinematic parameters of pregnant women during walking point to findings related to the first strategy. This suggests that, on the one hand, the woman increases her body stability in detriment of cost energy (based on kinematics) and, on the other hand, reduces the mechanical load in order to reduce the energy cost (based on kinetics). This reflection shows a balance between strategies, which require further analysis. This is in line with some theories of motor behavior that indicate that when the body is faced with constraints of the organism (and possibly other constraints) it self-organizes in order to have greater efficiency [21, 22].

With regard to changes during pregnancy, the morphological and body composition parameters are those that are widely known. However, it is not known how these variables influence the biomechanical parameters, or which of them give further explanation to mechanical changes in the musculoskeletal system of the pregnant woman. Thus, we suggest that future studies quantify the morphological and body composition changes and analyze how these changes influence the biomechanical parameters of women throughout pregnancy and in the postpartum period.

## 5. Conclusions

A descriptive longitudinal type design was performed, in which pregnant women were assessed in the first, second, and third trimesters of pregnancy and in the sixth month of the postpartum period. The biomechanical analysis of the lower limb during gait showed that pregnancy is a factor that influences the kinetic variables of the ankle, knee, and hip joints. The joint that undergoes the most evident changes is by far the hip. This fact may be justified by being closer to the body region with greater anatomical and morphological changes. The overall results point to biomechanical adjustments that show a decrease of the mechanical load of women throughout pregnancy, possibly in order to avoid strong interaction between women and the ground, confirmed by the decrease of GRF, the joint moments, and joint powers in the sagittal plane, whose parameters are primarily responsible for the progression of the body in space. These results point to some applications to this population, namely, the need to perform physical activities for the development of body stability, particularly the stability of the joints of the lower limbs.

## Figures and Tables

**Table 1:** Mean and standard deviation of the peak values for vertical, anterior-posterior, and medial-lateral components of the ground reaction forces.

Component	Side	Peaks	First trimester		Second trimester		Third trimester		Postpartum		*p*
			Mean	Std. Dev.	Mean	Std. Dev.	Mean	Std. Dev.	Mean	Std. Dev.	
Vertical	Right	**1st**	1.085	0.083	1.091	0.057	**1.073**	**0.057**	**1.110**	**0.100**	**(f) 0.013**
		**2nd**	0.797	0.063	0.811	0.057	**0.830**	**0.045**	**0.783**	**0.070**	**(f) 0.030**
		**3rd**	1.137	0.039	**1.126**	**0.044**	**1.086**	**0.030**	**1.156**	**0.042**	**(c) 0.026** **(f) 0.001**
	Left	1st	1.089	0.076	1.108	0.072	1.085	0.067	1.122	0.107	**ns**
		2nd	0.793	0.061	0.800	0.058	0.821	0.046	0.781	0.067	**ns**
		**3rd**	**1.136**	**0.045**	1.125	0.052	**1.089**	**0.037**	**1.147**	**0.050**	**(b) 0.001** **(f) 0.004**

Anterior-osterior	Right	**1st**	**−0.159**	**0.038**	−0.175	0.045	−0.161	0.032	**−0.182**	**0.050**	**(d) 0.044**
		2nd	0.207	0.031	0.219	0.024	0.204	0.028	0.215	0.035	** ns**
	Left	1st	−0.145	0.064	−0.173	0.027	−0.163	0.027	−0.182	0.042	** ns**
		**2nd**	0.187	0.046	0.208	0.025	**0.192**	**0.022**	**0.210**	**0.029**	**(f) 0.019**

Medial-lateral	Right	1st	−0.011	0.030	−0.034	0.039	−0.025	0.018	−0.017	0.032	** ns**
		2nd	−0.095	0.031	−0.086	0.029	−0.080	0.031	−0.094	0.033	** ns**
	Left	**1st**	−0.007	0.035	0.008	0.028	**0.027**	**0.022**	**−0.008**	**0.029**	**(f) 0.031**
		2nd	0.095	0.048	0.078	0.029	0.078	0.025	0.087	0.032	** ns**

(a) Significant differences between 1st and 2nd trimesters; (b) significant differences between 1st and 3rd trimesters; (c) significant differences between 2nd and 3rd trimesters; (d) significant differences between 1st trimester and PP; (e) significant differences between 2nd trimester and PP; (f) significant differences between 3rd trimester and PP; ns: nonsignificant.

**Table 2:** Mean and standard deviation of the joint moment peaks, and significance levels of the pairs of collections with significant changes for sagittal, frontal, and transverse planes.

Joint	Plane	Side	Peak	First trimester		Second trimester		Third trimester		Postpartum		*p*
				Mean	Std. deviation	Mean	Std. deviation	Mean	Std. deviation	Mean	Std. deviation	
Ankle	Sagittal	Right	1st	0.125	0.031	0.130	0.068	0.119	0.048	0.156	0.060	
			**2nd **	**−1.364**	**0.086**	−1.336	0.096	**−1.282**	**0.061**	−1.337	0.114	(b) 0.019
		Left	1st	0.150	0.056	0.127	0.052	0.103	0.038	0.162	0.057	
			**2nd **	−1.301	0.128	−1.340	0.084	**−1.294**	**0.107**	**−1.368**	**0.128**	(f) 0.015
	Frontal	Right	1st	0.349	0.170	0.459	0.153	0.376	0.162	0.364	0.191	
		Left	1st	0.311	0.176	0.309	0.177	0.382	0.195	0.377	0.231	
	Transverse	Right	1st	0.041	0.039	0.033	0.029	0.013	0.019	0.022	0.026	
			2nd	−0.132	0.054	−0.155	0.107	−0.136	0.038	−0.151	0.052	
		Left	1st	0.034	0.030	0.034	0.024	0.049	0.028	0.029	0.029	
			2nd	−0.161	0.093	−0.125	0.044	−0.097	0.056	−0.129	0.055	

Knee	Sagittal	Right	1st	0.341	0.314	0.364	0.368	0.366	0.254	0.502	0.371	
			2nd	−0.382	0.198	−0.339	0.169	−0.267	0.198	−0.294	0.186	
			3rd	0.171	0.091	0.209	0.138	0.173	0.111	0.249	0.166	
		Left	1st	0.355	0.425	0.347	0.365	0.428	0.310	0.476	0.487	
			2nd	−0.330	0.115	−0.364	0.130	−0.288	0.168	−0.361	0.109	
			3rd	0.219	0.087	0.169	0.121	0.186	0.099	0.185	0.101	

Hip	Sagittal	Right	**1st **	**−0.612**	**0.172**	−0.527	0.147	**−0.484**	**0.167**	**−0.618**	**0.202**	(b) 0.013 (f) 0.022
			**2nd **	**0.769**	**0.200**	**0.691**	**0.145**	**0.614**	**0.129**	**0.899**	**0.233**	(b) 0.031 (e) 0.009 (f) 0.001
		Left	**1st **	**−0.511**	**0.160**	−0.550	0.173	**−0.445**	**0.127**	**−0.598**	**0.155**	(d) 0.034 (f) 0.001
			2nd	0.789	0.141	0.687	0.209	0.653	0.074	0.798	0.254	
	Frontal	Right	1st	−0.870	0.197	−0.943	0.291	−0.856	0.097	−0.828	0.143	
			2nd	−0.735	0.146	−0.755	0.217	−0.759	0.133	−0.688	0.160	
		Left	1st	−0.803	0.113	−0.877	0.172	−0.854	0.097	−0.836	0.169	
			2nd	−0.735	0.145	−0.802	0.178	−0.715	0.148	−0.721	0.140	
	Transverse	Right	**1st **	**−0.237**	**0.057**	**−0.441**	**0.363**	**−0.342**	**0.071**	**−0.257**	**0.083**	(a) 0.006 (b) 0.008 (e) 0.013
		Left	1st	−0.255	0.051	−0.317	0.079	−0.301	0.110	−0.260	0.086	

(a) Significant differences between 1st and 2nd trimesters; (b) significant differences between 1st and 3rd trimesters; (c) significant differences between 2nd and 3rd trimesters; (d) significant differences between 1st trimester and PP; (e) significant differences between 2nd trimester and PP; (f) significant differences between 3rd trimester and PP; ns: nonsignificant. Units are in N·m·Kg^−1^.

**Table 3:** Mean and standard deviation of the joint power peaks, and significance levels of the pairs of collections with significant changes for sagittal, frontal, and transverse planes.

Joint	Plane	Side	Peak	First trimester		Second trimester		Third trimester		Postpartum		*p*
				Mean	Std. deviation	Mean	Std. deviation	Mean	Std. deviation	Mean	Std. deviation	
Ankle	Sagittal	Right	1st	−0.720	0.200	−0.644	0.200	−0.624	0.195	−0.613	0.274	
			2nd	3.003	0.830	2.884	0.701	2.647	0.674	2.987	0.889	
		Left	1st	−0.654	0.287	−0.668	0.269	−0.604	0.273	−0.638	0.339	
			2nd	2.771	0.962	2.846	0.672	2.465	0.726	2.984	0.993	
	Frontal	Right	1st	−0.081	0.058	−0.119	0.077	−0.074	0.046	−0.090	0.099	
			2nd	0.347	0.196	0.551	0.314	0.436	0.295	0.446	0.367	
		Left	1st	−0.062	0.034	−0.075	0.093	−0.089	0.043	−0.078	0.037	
			2nd	0.314	0.288	0.323	0.245	0.462	0.403	0.586	0.451	
	Transverse	Right	1st	−0.040	0.040	−0.086	0.176	−0.032	0.053	−0.032	0.021	
			2nd	−0.119	0.049	−0.146	0.093	−0.114	0.075	−0.134	0.075	
		Left	1st	−0.048	0.031	−0.050	0.022	−0.040	0.018	−0.049	0.030	
			**2nd**	**−0.150**	**0.076**	**−0.148**	**0.065**	**−0.087**	**0.066**	−0.139	0.101	(b) 0.030 (c) 0.025

Knee	Sagittal	Right	1st	−0.525	0.412	−0.521	0.577	−0.546	0.425	−0.701	0.581	
			**2nd**	**0.345**	**0.299**	0.349	0.322	0.276	0.156	**0.490**	**0.377**	(d) 0.006
			3rd	−0.673	0.178	−0.782	0.523	−0.566	0.223	−0.829	0.265	
		Left	1st	−0.481	0.623	−0.476	0.458	−0.587	0.523	−0.621	0.891	
			2nd	0.457	0.391	0.374	0.270	0.390	0.221	0.592	0.530	
			3rd	−0.791	0.306	−0.678	0.332	−0.585	0.154	−0.714	0.275	

Hip	Sagittal	Right	1st	0.435	0.267	0.400	0.203	0.478	0.272	0.407	0.325	
			**2nd**	−0.749	0.322	−0.675	0.267	**−0.614**	**0.239**	**−0.987**	**0.474**	(f) 0.027
		Left	1st	0.339	0.183	0.537	0.267	0.444	0.184	0.402	0.246	
			**2nd**	−0.744	0.257	−0.672	0.310	**−0.610**	**0.194**	**−0.867**	**0.338**	(f) 0.014
	Frontal	Right	1st	−0.592	0.211	−0.693	0.310	−0.619	0.255	−0.654	0.272	
			2nd	0.736	0.319	0.720	0.344	0.570	0.231	0.704	0.261	
			3rd	0.634	0.153	0.642	0.302	0.721	0.189	0.632	0.259	
		Left	1st	−0.493	0.256	−0.635	0.372	−0.600	0.236	−0.586	0.274	
			2nd	0.655	0.219	0.740	0.267	0.596	0.125	0.828	0.347	
			3rd	0.670	0.284	0.767	0.222	0.648	0.197	0.595	0.189	
	Transverse	Right	**1st**	**−0.306**	**0.140**	**−0.670**	**0.776**	−0.414	0.196	−0.354	0.199	(a) 0.008
			2nd	0.061	0.041	0.115	0.117	0.068	0.103	0.067	0.046	
		Left	1st	−0.330	0.111	−0.555	0.308	−0.394	0.199	−0.453	0.247	
			2nd	0.062	0.043	0.135	0.178	0.052	0.050	0.090	0.064	

(a) Significant differences between 1st and 2nd trimester; (b) significant differences between 1st and 3rd trimesters; (c) significant differences between 2nd and 3rd trimesters; (d) significant differences between 1st trimester and PP; (e) significant differences between 2nd trimester and PP; (f) significant differences between 3rd trimester and PP; ns: nonsignificant. Units are in W·Kg^−1^.
